# Protracted encephalopathy and subacute combined degeneration associated with chronic nitrous oxide use: a case report

**DOI:** 10.3389/fpsyt.2026.1797631

**Published:** 2026-05-15

**Authors:** Kaevon Brasfield, Eugene Pahk, Conrado Sevilla

**Affiliations:** Department of Psychiatry, Mission Community Hospital, Panorama City, CA, United States

**Keywords:** B12, delirium, encephalopathy, nitrous oxide, subacute combined degeneration, inhalant use disorder, addiction

## Abstract

Nitrous oxide is a dissociative hallucinogen that is increasingly used recreationally, in part due to its widespread availability. Its use is known to cause subacute combined degeneration via inactivation of vitamin B12; it may also result in acute delirium and chronic progressive encephalopathy. Though current practice guidelines call for treatment of neurological sequelae of nitrous oxide use with vitamin B12 supplementation, a paucity of long-term outcome data limits our ability to guide extended courses of treatment. In this report, we discuss a case of protracted encephalopathy associated with nitrous oxide use. We track the response to vitamin B12 supplementation in the hospital setting using the Mini-Mental Status Exam to assess the severity and improvement of cognitive impairment. We also review the patient’s comorbid medical and psychiatric conditions, which complicate diagnosis and treatment planning in this patient population.

## Introduction

Public health data suggest an increasing prevalence of substance use disorders, particularly in the United States (1). Nitrous oxide, a short-acting analgesic used in some healthcare settings, is inhaled recreationally for its dissociative hallucinogenic properties. Its wide availability may contribute to its popularity; the 2021 Global Drug Survey reported that 10% of United States respondents had used the substance in the past year (2). Though recreational nitrous oxide was previously associated with raves and music festivals, recent public health data suggest that home use has become increasingly common (3).

Chronic nitrous oxide use can result in subacute combined degeneration, a condition characterized by peripheral neuropathy, myelopathy, and cognitive impairment (4). This occurs via inactivation of vitamin B12 (cobalamin), preventing it from acting as a cofactor in myelin synthesis. Impaired DNA synthesis and homocysteine-mediated neurotoxicity may also contribute to neurologic impairment associated with cobalamin deficiency (5). Signs may include hyperreflexia and hyporeflexia, paresthesia, numbness, lower-limb motor deficits, and proprioceptive ataxia. Cognitive impairment and psychotic symptoms are also known to be associated with cobalamin deficiency (6). A recent narrative review has established that nitrous oxide-associated psychiatric symptoms, even in the absence of neurologic deficits, can occur and may be underrecognized (7).

The expected chronicity of cognitive impairment that may result from chronic nitrous oxide use is not well understood. Though some case reports describe encephalopathy associated with nitrous oxide use (8–10), long-term outcome data are scarce. Cobalamin supplementation in cases of suspected subacute combined degeneration has been described in the literature (11). The efficacy of this intervention in reversing the syndrome’s cognitive symptoms is unclear, with many patients experiencing incomplete symptom resolution (11). Other mechanisms of neurotoxicity associated with nitrous oxide abuse (e.g., repeated inhalant-induced hypoxemia) may play a role in addition to cobalamin inactivation (12), implying limitations in the utility of vitamin supplementation.

As the rate of inhalant use increases, further research on the prognosis of nitrous oxide-induced cognitive impairment and its potential response to cobalamin supplementation could guide future treatment decisions. The following report describes a case of severe cognitive impairment in a young adult who uses nitrous oxide, her response to cobalamin supplementation, and a discussion of comorbid and predisposing factors.

## Clinical presentation

The patient is a 20-year-old woman with a history of polysubstance use. She presented to the emergency department of a hospital in Los Angeles County with right leg pain, lower extremity weakness, and altered mental status. History was obtained from the patient’s brother, as she was unable to provide information due to severe attentional impairment. She was reported to have inhaled an unknown quantity of nitrous oxide on a daily basis. A urine drug-of-abuse panel was negative for amphetamines, barbiturates, benzodiazepines, cannabinoids, cocaine, opiates, and phencyclidine. Serum ethanol levels were undetectable, and no evidence of recent alcohol use was reported. Urine ethyl glucuronide was not tested.

Medical history was notable for two prior episodes of deep vein thrombosis requiring hospitalization and anticoagulation treatment. Several prior cosmetic surgeries, in addition to N_2_O use, were believed by her previous providers to confer a coagulation risk. Psychosocial history included chronic physical and sexual abuse beginning in early childhood, followed by entry into the foster care system at age 11. She had completed secondary school; there was no known history of intellectual or developmental disability.

Prior substance use included insufflated heroin and cocaine, and alcohol use two to three times weekly in the past. In the year prior to this presentation, her use of nitrous oxide increased as her use of other substances ceased. The patient estimated using up to 20 handheld canisters of inhaled nitrous oxide daily (weight in ounces not recalled). In the weeks before hospital admission, she became unable to ambulate independently and required assistance with activities of daily living. Initial differential diagnosis included nitrous oxide intoxication, deep vein thrombosis, subacute combined degeneration, alcohol withdrawal syndrome, and infectious causes of encephalopathy or myelopathy.

## Physical, psychiatric, and neurologic exam findings

Physical examination in the emergency department identified erythematous and purulent cold burn marks, believed to be cold burn injuries sustained from contact with a nitrous oxide canister during use. Tenderness in the suprapubic area and bilateral edema of the lower extremities were also noted. She was described as anxious-appearing. The neurological examination was notable for symmetric lower extremity weakness and reduced sensation to light touch. No cranial nerve deficits were appreciated.

On initial psychiatric assessment the following hospital day, mental status examination revealed an age-appearing woman disoriented to date and season. Attentional impairment limited her engagement in conversation. Her thought process was grossly disorganized, and insight into her condition was impaired. She endorsed passive suicidal ideation but articulated no intent to die or plan to end her life. Affect was euthymic, incongruent with suicidal thought content. She denied auditory, visual, and somatic perceptual disturbances. A Mini-Mental State Examination (MMSE) was conducted at the bedside and scored 13 out of 30, suggesting severe cognitive impairment.

## Laboratory and imaging findings

A complete blood count was notable for macrocytic anemia. Leukocytosis, fever, and hypotension were absent. Prothrombin time and international normalized ratio were within normal limits on admission; activated partial thromboplastin time was elevated at 56.3 s. Screening tests for factor V Leiden, antiphospholipid antibody syndrome, and abnormalities in proteins C and S and antithrombin III were unremarkable. In the emergency department, a computerized tomography (CT) scan of the head as well as CT of the chest, abdomen, and pelvis revealed no acute pathology. Magnetic resonance imaging (MRI) studies were recommended but not conducted during this episode of hospitalization. Several months after discharge, the patient underwent noncontrast MRI of the brain and cervical, thoracic, and lumbar spinal regions. No acute or chronic abnormalities were reported, though the brain MRI and cervical spine MRI were noted to be degraded by motion artifact.

Serum vitamin B12 concentration was low at 150 pg/mL on admission; serum methylmalonic acid was significantly elevated at 2,927 nmol/L, as was homocysteine at 33.5 μmol/L. Serum thiamine, folate, and ammonia were within reference ranges. Rapid plasma reagin and serum treponemal assays did not detect evidence of treponemal infection. There was a mild elevation in alanine aminotransferase, with aspartate aminotransferase within the reference range. Blood urea nitrogen, creatinine, phosphorus, and magnesium concentrations were mildly elevated.

## Therapeutic interventions and course

**Figure 1:**
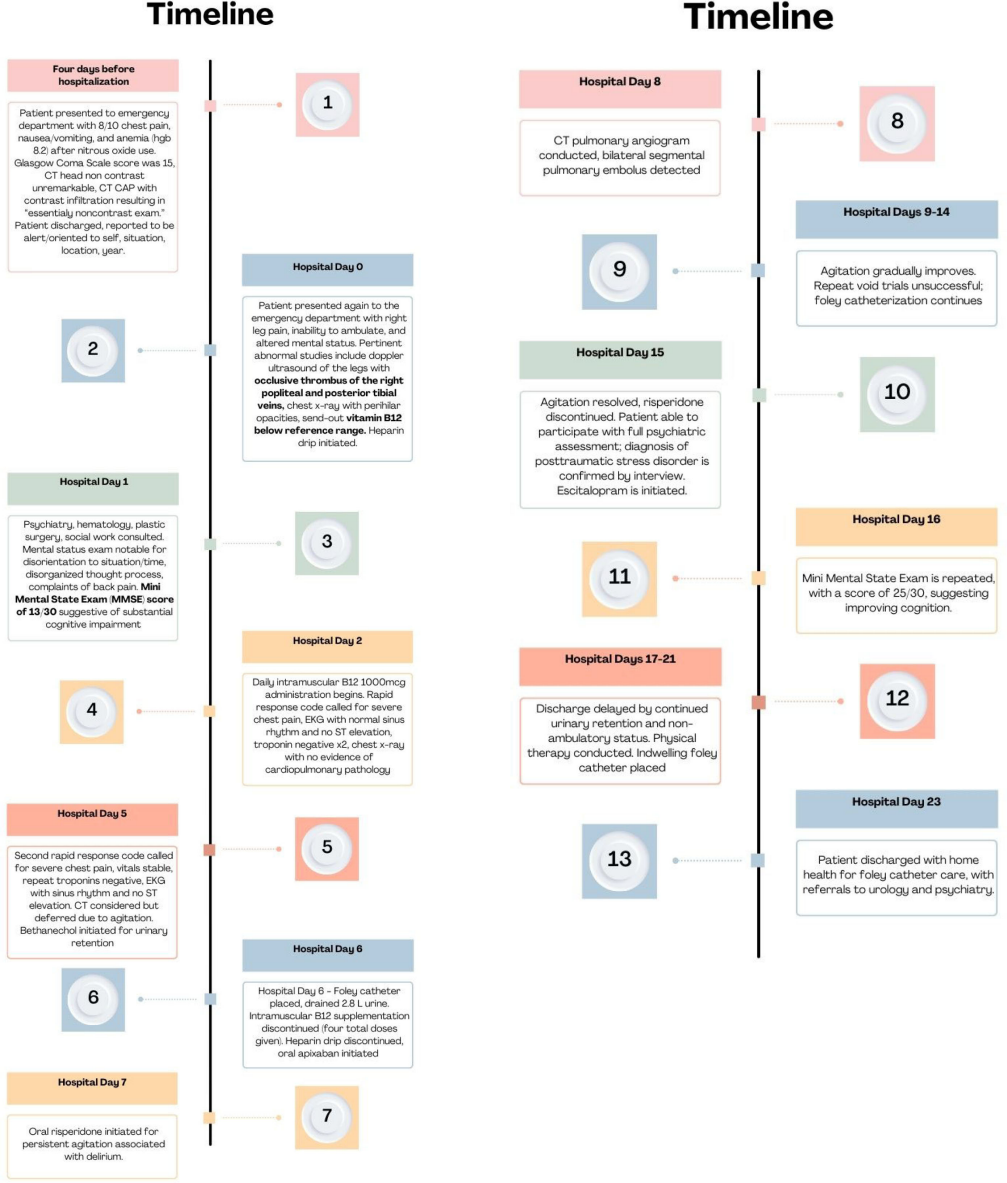
Timeline of hospital course.

Full hospital course is summarized in **Figure 1**. Empiric cefazolin was initiated in the emergency department, and plastic surgery was consulted to evaluate the patient’s soft tissue infection of the lower extremities. Surgical intervention was deferred due to the patient’s severe encephalopathy. Multimodal analgesics were used for pain. A heparin drip was initiated for deep vein thrombosis; intravenous fluids were administered for acute kidney injury. Due to worsening chest pain on hospital day nine, a CT pulmonary angiogram was obtained and revealed a pulmonary embolus.

Psychiatry was consulted for the management of intermittent behavioral disturbances. Haloperidol intramuscular injections and soft restraints were used as needed for severe behavioral agitation (yelling, removing intravenous lines, attempting to ambulate despite profound lower extremity weakness). Due to frequent use of intramuscular haloperidol and restraints, orally disintegrating risperidone tablets were administered at a dose of 0.5 mg twice daily. This coincided with a reduction in episodes of behavioral agitation.

Vitamin B12 was supplemented, first via intramuscular and then oral administration. Upon discharge, serum vitamin B12 was within the reference range at 616 pg/mL. Throughout the hospitalization, scores on repeat Mini-Mental State Examinations (MMSEs) increased from 13/30 on hospital day 2 to 17/30 on hospital day 9. On hospital day 14, oral risperidone was discontinued without recurrence of behavioral disturbances. On hospital day 16, the MMSE score improved to 25/30. No evidence of hallucinations or delusions was detected throughout the hospitalization. In a discussion of her psychosocial history that occurred after improvement in her delirium, the patient’s disclosure of exposure to a traumatic stressor prompted an evaluation for posttraumatic stress disorder (PTSD). The patient was diagnosed with PTSD, and treatment with escitalopram was initiated.

Discharge was delayed due to neurogenic urinary retention, the treatment of which was associated with resolution of acute kidney injury. The patient was discharged with an indwelling Foley catheter and home health services for catheter maintenance. She was referred to peer support groups for substance use disorders and to an outpatient psychiatry clinic for treatment of PTSD but was lost to follow-up.

## Discussion

Though delirium due to nitrous oxide intoxication was considered the most likely explanation for her presenting condition, the complexity of our patient’s case presented significant diagnostic challenges. A comorbid component of alcohol withdrawal syndrome could not initially be ruled out; after several days of observation, this was deemed less probable given low Clinical Alcohol Withdrawal Assessment (CIWA) scores and poor response to benzodiazepines. A urine ethyl glucuronide test could have strengthened this assessment. The delirium was likely exacerbated by comorbid medical conditions, including pulmonary embolus, urinary retention, soft tissue infection, acute kidney injury, and anemia. Hospital delirium was considered a contributing factor; however, the severity of confusion on presentation, as well as the patient’s improvement in response to cobalamin supplementation, suggested that subacute combined degeneration was the primary causative process.

Given our inability to quantify the recency and amount of nitrous oxide use, a distinction between intoxication and withdrawal delirium could not be readily made. To the authors’ knowledge, no case reports have specifically described a withdrawal syndrome associated with nitrous oxide. This patient’s prolonged course of confusion, which demonstrated a delayed response to cobalamin supplementation, raises the question of whether heavy, chronic users of nitrous oxide can experience delirium associated with withdrawal. This is a salient question for clinical practice, given increasing rates of nitrous oxide use in the public.

Our patient’s case also illustrates the often multifactorial nature of acute cognitive impairment that can occur in patients who use nitrous oxide. Vitamin B12 deficiency was one probable encephalopathic factor; findings of elevated methylmalonic acid and homocysteine were suggestive of recent and frequent N_2_O use (13), supporting an association between our patient’s cobalamin deficiency and recent N_2_O use. Additional putative mechanisms for N_2_O-mediated cognitive impairment, namely the possibility of repeated hypoxic brain injury sustained during inhalation of the drug (12, 14), may also have contributed to the presentation. Given the prothrombotic effect of nitrous oxide, delirium due to pulmonary embolus or transient cerebral ischemia must also be considered. Interventions aimed at reversing or preventing cobalamin deficiency may not adequately address cognitive impairment caused by these other mechanisms of toxicity. Further research is needed to disentangle the various contributors to cognitive impairment associated with habitual N_2_O use. This research would aid in prognostic assessment and in characterizing the efficacy of interventions such as long-term cobalamin supplementation, as well as provide clinical guidance in the use of laboratory measures such as homocysteine and methylmalonic acid levels for monitoring treatment progress.

Other factors limit our ability to establish a causal relationship between cobalamin supplementation and improvement in acute cognitive impairment in this patient. The Mini-Mental State Examination is subject to a practice effect (15), raising concern that improved scores on repeated administrations may not reflect global improvement in cognition. Furthermore, this examination is intended for the assessment of chronic cognitive impairment; a standardized measure for assessing delirium would have been more appropriate. Several important examinations were recommended by medical consultants but not carried out during the patient’s hospitalization—namely, brain MRI, lumbar puncture, and electroencephalography. Tests for other GABAergic agents that are associated with withdrawal syndromes, such as gabapentin, baclofen, and gamma-hydroxybutyric acid, would also have been appropriate. Though the patient’s improving clinical status with administration of cobalamin was reassuring, these additional studies could have ruled out other contributing factors to cognitive impairment.

Our patient’s prior use of other psychoactive substances may also have impacted her cognition via a myriad of mechanisms. Polysubstance use may be common in people who use nitrous oxide; a CDC publication reviewing surveillance data from poison control centers over a four-year period indicated that 30% of cases involving nitrous oxide also involved other substances (16). Previous episodes of delirium have been shown to be associated with long-term cognitive impairment (17); it is possible that prior undetected episodes of delirium related to intoxication or withdrawal may have contributed to our patient’s protracted course of illness.

The identification of our patient’s chronic posttraumatic stress disorder highlights the utility of a prompt and comprehensive psychiatric assessment for patients who seek care for substance use disorders and their sequelae. Professional guidelines recommend screening for posttraumatic stress disorder in patients with substance use disorder and treatment with trauma-informed psychotherapy when the conditions co-occur (18). Systematic reviews suggest a high prevalence of comorbidity between depressive disorders and substance use disorders (19). Epidemiological data have provided strong evidence of an association between adverse childhood experiences and subsequent substance use disorders (20), underscoring the relevance of psychosocial data for risk stratification and treatment planning. To address the increasing prevalence of nitrous oxide use, future preventive care efforts may emphasize early education and psychiatric intervention in populations at high risk for the development of inhalant use disorder.

## Conclusion

In this case report, we present the clinical course of a patient admitted with deep vein thrombosis and delirium due to nitrous oxide intoxication; neurological findings were suggestive of subacute combined degeneration. We discussed case factors that may have contributed to the patient’s protracted course of delirium, including comorbid medical conditions, a history of polysubstance use, and untreated posttraumatic stress disorder.

Though our patient’s delirium improved considerably over the course of several weeks, during which time vitamin B12 was supplemented, we are unable to confidently attribute her cognitive improvement to this intervention given the many medical and psychiatric comorbidities identified. Further research is needed to identify the expected response of nitrous oxide-induced encephalopathy to vitamin B12 supplementation, identify biomarkers of treatment progress, and better understand long-term cognitive outcomes associated with chronic nitrous oxide use.

## Patient perspective

Due to the patient’s protracted course of encephalopathy and myelopathy, this section comprises the authors’ synthesis of the patient’s statements throughout this hospitalization as documented in the medical record. She reported pain “in my whole body” and called for her mother repeatedly on the first hospital day. She described feeling “delusional” and did not feel comfortable making medical decisions for herself. As her agitation waned, she told providers that she used nitrous oxide to “not feel anything anymore.”

Over the course of the hospitalization, she began to feel “less sad” and less confused. She disclosed a history of physical and sexual abuse dating back to early childhood, which led to placement in the foster care system. She briefly received psychiatric care in her early adolescence but was lost to follow-up. Our patient recalls being constantly “on-go-mode” and framed her substance use as a way of “distracting” herself from intrusive memories. Though she reported feeling “happy” toward the end of the hospitalization, she expressed apprehension regarding psychosocial stressors that pose continued challenges for her recovery.

## Data Availability

The original contributions presented in the study are included in the article/supplementary material. Further inquiries can be directed to the corresponding author.
